# Activity of cefepime, carbapenems and new β-lactam/β-lactamase inhibitor combinations on *Enterobacter cloacae* complex and *Klebsiella aerogenes* in Spain (SMART 2016–2022)

**DOI:** 10.1093/jacamr/dlae087

**Published:** 2024-06-06

**Authors:** Ángel Rodríguez-Villodres, Esperanza Lepe-Balsalobre, José Manuel Ortiz De La Rosa, Salvador Giner Almaraz, Elisa González De Herrero, Emilia Cercenado, Sergio García-Fernández, Rafael Benito, Ricardo Ponz Mir, Rafael Cantón, José Antonio Lepe

**Affiliations:** Clinical Unit of Infectious Diseases, Microbiology and Parasitology, University Hospital Virgen del Rocío, Seville, Spain; Institute of Biomedicine of Seville (IBiS), University Hospital Virgen del Rocío/CSIC/University of Seville, Seville, Spain; Centro de Investigación Biomédica en Red de Enfermedades Infecciosas (CIBERINFEC), Instituto de Salud Carlos III, Madrid, Spain; Servicio de Laboratorio, Hospital de Riotinto, Huelva, Spain; Clinical Unit of Infectious Diseases, Microbiology and Parasitology, University Hospital Virgen del Rocío, Seville, Spain; Institute of Biomedicine of Seville (IBiS), University Hospital Virgen del Rocío/CSIC/University of Seville, Seville, Spain; Centro de Investigación Biomédica en Red de Enfermedades Infecciosas (CIBERINFEC), Instituto de Salud Carlos III, Madrid, Spain; Servicio de Microbiología, Hospital Universitari y Politécnic La Fe, Valencia, Spain; Centro de Investigación Biomédica en Red de Enfermedades Infecciosas (CIBERINFEC), Instituto de Salud Carlos III, Madrid, Spain; Servicio de Microbiología, Hospital Son Espases, IdISBa, CIBERINFEC, Palma de Mallorca, Mallorca, Spain; Servicio de Microbiología Clínica y Enfermedades Infecciosas, Hospital Universitario Gregorio Marañón, Madrid, Spain; Centro de Investigación Biomédica en Red de Enfermedades Respiratorias (CIBERES), Instituto de Salud Carlos III, Madrid, Spain; Centro de Investigación Biomédica en Red de Enfermedades Infecciosas (CIBERINFEC), Instituto de Salud Carlos III, Madrid, Spain; Servicio de Microbiología, Hospital Universitario Marqués de Valdecilla-IDIVAL, Santander, Spain; Hospital Clínico Universitario Lozano Blesa, Zaragoza, Departamento de Microbiología, Universidad de Zaragoza, Instituto de Investigación Sanitaria de Aragón, Zaragoza, Spain; Departamento Médico, MSD, Madrid, Spain; Centro de Investigación Biomédica en Red de Enfermedades Infecciosas (CIBERINFEC), Instituto de Salud Carlos III, Madrid, Spain; Servicio de Microbilogía, Hospital Universitario Ramón y Cajal and Instituto Ramón y Cajal de Investigación Sanitaria (IRYCIS), Madrid, Spain; Clinical Unit of Infectious Diseases, Microbiology and Parasitology, University Hospital Virgen del Rocío, Seville, Spain; Institute of Biomedicine of Seville (IBiS), University Hospital Virgen del Rocío/CSIC/University of Seville, Seville, Spain; Centro de Investigación Biomédica en Red de Enfermedades Infecciosas (CIBERINFEC), Instituto de Salud Carlos III, Madrid, Spain; Department of Microbiology, University of Seville, Seville, Spain

## Abstract

**Objectives:**

To analyse the susceptibility profile to cefepime, carbapenems and new β-lactam/β-lactamase inhibitor combinations in *Enterobacter cloacae* complex and *Klebsiella aerogenes* isolated from intra-abdominal, urinary, respiratory and bloodstream infections in the SMART (Study for Monitoring Antimicrobial Resistance Trends) surveillance study in Spain.

**Methods:**

The susceptibilities of 759 isolates (473 *E. cloacae* complex and 286 *K. aerogenes*) collected in 11 Spanish hospitals from 2016 to 2022 were analysed following the EUCAST 2023 criteria. Molecular characterization looking for β-lactamase genes was performed through PCR and DNA sequencing analysis.

**Results:**

*E. cloacae* complex showed resistance to third-generation cephalosporins in 25% of the cases, whereas *K. aerogenes* was resistant in 35%. Regarding cefepime, resistance in *E. cloacae* was higher (10%) than in *K. aerogenes* (2%). Carbapenems showed >85% activity in both microorganisms. Ceftazidime/avibactam, imipenem/relebactam and meropenem/vaborbactam had good activity against these microorganisms (>95%). In contrast, the activity of ceftolozane/tazobactam was lower (80%). A high proportion of the isolates resistant to new β-lactam/β-lactamase inhibitor combinations carried a carbapenemase, mainly OXA-48-like and VIM-1.

**Conclusions:**

Ceftazidime/avibactam, imipenem/relebactam and meropenem/vaborbactam show high activity against both *E. cloacae* complex and *K. aerogenes* isolates recovered in the SMART-Spain study. In contrast, differences have been found in the case of cefepime, showing more activity against *K. aerogenes* than *E. cloacae* complex. These results are useful for antimicrobial stewardship programmes and for the implementation of local and national guidelines.

## Introduction

Antimicrobial resistance is a major problem worldwide. A recent study has estimated that there were 4.95 million deaths associated with bacterial antimicrobial resistance (AMR), in 2019 alone.^1^ Of these, 1.27 million deaths were attributable directly to AMR. Another study from the ECDC estimated that, in 2015, there were 671 689 infections by MDR bacteria, causing 33 000 deaths.^2^ Remarkably, more than 50% were healthcare-related infections. These types of infections have a major clinical impact because they affect patients with severe comorbidities, in which management is difficult, increasing mortality.^3^

In this sense, surveillance programmes can help to develop antimicrobial stewardship programmes, inform treatment decisions, guide national and local policies and clinical guidelines, and direct efforts to develop new treatment options, improving the clinical outcome of the patients.^4^ The Study for Monitoring Antimicrobial Resistance Trends (SMART) is one of the largest and longest-standing AMR surveillance programmes. It has been operating since 2002, monitoring trends of antimicrobial susceptibility of aerobic and facultative Gram-negative bacilli from intra-abdominal infections, urinary tract infections, lower-respiratory tract infections and bloodstream infections.^4,5^

In recent years, *Enterobacter* species and *Klebsiella aerogenes* (formerly *Enterobacter aerogenes*) have acquired relevance among the microorganisms causing healthcare-related infections due to their ability to develop AMR.^6^ The presence of an inducible chromosomal AmpC β-lactamase confers extended resistance to third-generation cephalosporins (such as ceftazidime, ceftriaxone or cefotaxime) and piperacillin/tazobactam, when it is overexpressed, limiting the therapeutic options for patients.^7^

Cefepime is the antibiotic of choice to treat infections caused by these type of microorganisms because of its low ability to induce AmpC β-lactamases.^8^ Carbapenems also are considered to treat infections by these microorganisms.^8^ The IDSA recommends using carbapenems when MICs of cefepime are higher than 2 mg/L due to the high risk of co-producing an ESBL enzyme.^8^ However, some studies have reported resistance to both cefepime and carbapenems in *E. cloacae* and *K. aerogenes.*^9–11^ New β-lactam/β-lactamase inhibitor combinations, such as ceftazidime/avibactam, imipenem/relebactam or meropenem/vaborbactam, also could be alternative options for the treatment of *Enterobacter* spp. and/or *K. aerogenes* infections, especially when they are hyperproducing AmpC and exhibit carbapenem resistance that is not due to MBLs.^8^

Despite these recommendations, there are few recent studies monitoring the activity of these antibiotics against *Enterobacter* spp. and *K.* aerogenes.^12,13^ In this study, we analysed the susceptibility trend to cefepime, carbapenems and new β-lactam/β-lactamase inhibitor combinations in *Enterobacter* spp. and *K. aerogenes* during a 7 year period (2016–2022), focusing also on the molecular profile associated with resistance to these antibiotics.

## Material and methods

### Bacterial isolates and antimicrobial susceptibility testing

Clinical isolates of *E. cloacae* complex and *K. aerogenes* were recovered at 11 Spanish hospitals from 2016 to 2022. Isolates were subsequently shipped to a central laboratory (IHMA, Schaumburg, IL, USA) for identification by MALDI-TOF and antimicrobial susceptibility testing (AST) by broth microdilution, following the standard ISO recommendations. The following antibiotics were tested: piperacillin/tazobactam, ceftazidime, ceftriaxone, cefepime, ertapenem, imipenem, meropenem, ceftolozane/tazobactam, ceftazidime/avibactam, imipenem/relebactam and meropenem/vaborbactam. The EUCAST 2023 clinical breakpoints were used to analyse the susceptibility/resistance criteria. To see if there is a trend in the susceptibility profile over the years, we ran a weighted least squares analysis accounting for serial data autocorrelation with Joinpoint Regression v.5.1.0. (Statistical Methodology and Applications Branch, Surveillance Research Program, National Cancer Institute, USA). The time trends for each variable were analysed to find whether the slope significantly increased or decreased over time, or on the contrary, stayed steady over time, which indicates no significant differences among all the annual data in the series. We used a first-order autocorrelation parameter estimated from the data with a permutation test as the model selection method.^14^

### Molecular characterization

Molecular testing was also centralized at IHMA and included all the isolates resistant to at least one of the following antibiotics: imipenem, imipenem/relebactam or ceftolozane/tazobactam. Molecular testing consisted of screening the following β-lactamase genes through PCR and DNA sequencing: class A ESBLs (TEM, SHV, CTX-M, VEB, PER and GES); class C plasmid AmpC (ACC, ACT, CMY, DHA, FOX, MIR and MOX) and carbapenemases (KPC, GES, NDM, IMP, VIM, GIM, SPM and OXA-48-like), as previously described.^15,16^

## Results

### Clinical isolates features

A total of 759 isolates encompassing 473 (62.3%) *E. cloacae* complex and 286 (37.7%) *K. aerogenes* were included in the study. These microorganisms were isolated mainly from respiratory samples (*n* = 366), followed by intra-abdominal (*n* = 182), blood (*n* = 76) and urinary tract (*n* = 63) (Table S1, available as Supplementary data at *JAC-AMR* Online).

### Susceptibility profile to classic antibiotics

Table 1 shows the susceptibility profile of both microorganisms. Around 75% of the *E. cloacae* complex and 65% of the *K. aerogenes* isolates were susceptible to third-generation cephalosporins and piperacillin/tazobactam (Table 1). Regarding cefepime, there were notable differences between the microorganisms, despite it having good activity against both: *E. cloacae* complex remained less susceptible (90%) than *K. aerogenes* (98%). Overall, carbapenems (ertapenem, imipenem and meropenem) showed very good activity (>90%) whereas ertapenem activity was lower (86%) for *E. cloacae* complex (Table 1).

**Table 1.** Weighted least squares regression trend analysis of the percent susceptibility profile in *Enterobacter cloacae* complex and *Klebsiella aerogenes*

			% Susceptibility										
Microorganism	Year	Number of isolates	TZP	CAZ	CRO	FEP	ETP	IPM	MEM	C/T	CZA	IPM/REL	MEM/VAB
*Enterobacter cloacae*	2016	117	69	65	63	90	83	97	98	79	—	99	—
	2017	54	77	75	72	84	85	94	99	79	—	94	—
	2018	70	81	77	74	93	87	95	95	85	100	95	—
	2019	66	72	66	65	91	84	97	97	80	97	98	98
	2020	44	68	65	59	89	81	93	93	77	95	93	97
	2021	43	69	65	65	93	81	97	97	74	100	97	100
	2022	79	82	82	81	91	90	95	94	84	95	95	98
	**Total**	**473**	**75**	**75**	**72**	**90**	**86**	**97**	**97**	**81**	**97**	**97**	**97**
		Trend slope (SE)	**1.16 (1.08)**	**1.33 (1.17)**	**1.91 (1.27)**	**0.66 (0.28)**	**0.63 (0.50)**	**0.08 (0.10)**	**−0.64 (0.35)**	**−0.27 (0.69)**	**−0.39 (0.55)**	**−0.14 (0.25)**	**0.67 (0.22)**
		***P* value**	**0.332**	**0.304**	**0.192**	**0.068**	**0.261**	**0.487**	**0.127**	**0.714**	**0.532**	**0.604**	**0.091**
*Klebsiella aerogenes*	2016	58	69	65	67	100	98	100	100	86	—	100	—
	2017	40	72	65	67	95	95	95	95	85	—	95	—
	2018	28	57	53	64	100	100	100	100	82	100	100	—
	2019	37	64	64	62	100	91	94	97	78	100	100	100
	2020	41	58	56	56	95	85	92	95	75	100	100	100
	2021	52	57	57	57	100	94	94	94	82	98	100	100
	2022	30	80	73	73	97	96	100	100	93	100	100	100
	**Total**	**286**	**65**	**68**	**64**	**98**	**94**	**91**	**99**	**83**	**99**	**99**	**100**
		Trend slope (SE)	**−0.19 (1.55)**	**0.13 (1.08)**	**−0.35 (1.23)**	**0.01 (0.27)**	**−0.82 (0.95)**	**−0.42 (0.60)**	**−0.34 (0.35)**	**0.68 (1.46)**	**−0.36 (0.15)**	**0.49 (0.22)**	**0.00 (0.00)**
		***P* value**	**0.907**	**0.91**	**0.785**	**0.975**	**0.426**	**0.509**	**0.365**	**0.663**	**0.095**	**0.077**	**0.99**

CAZ, ceftazidime; CRO, ceftriaxone; C/T, ceftolozane/tazobactam; CZA, ceftazidime/avibactam; ETP, ertapenem; FEP, cefepime; IPM, imipenem; IPM/REL, imipenem/relebactam; MEM, meropenem; MEM/VAB, meropenem/vaborbactam; SE, standard error; TZP, piperacillin/tazobactam.

### Activity of new β-lactam/β-lactamase inhibitor combinations

Ceftazidime/avibactam, imipenem/relebactam and meropenem/vaborbactam showed good activity against *E. cloacae* complex and *K. aerogenes* clinical isolates (>95%). Furthermore, this activity had remained stable over recent years (Table 1). In contrast, ceftolozane/tazobactam had less activity against both genera of microorganisms (around 80%).

### Molecular characterization

#### E. cloacae complex

The molecular resistance profile for the *E. cloacae* complex is presented in Table 2. In summary, 47 of the 473 isolates (10%) were resistant to cefepime. Of these, 26 (55.3%) harboured a carbapenemase gene, 3 (6.4%) an ESBL gene, and another 3 (6.4%) a combination of an ESBL plus a plasmidic AmpC β-lactamase. In the remaining isolates (*n* = 15, 31.9%) no β-lactamases were found, except for the chromosomal *ampC* gene encoding the AmpC β-lactamase, characteristic of these microorganisms. Eighteen isolates showed resistance to imipenem and 23 to meropenem. Among these, one and one isolates, respectively, did not carry any carbapenemase gene (Table 2).

**Table 2.** Molecular profiles associated with resistant isolates of *Enterobacter cloacae*

Microorganism	Total isolates (*n*)	FEP	IPM	MEM	C/T	CZA	IPM/REL	MEM/VAB
*Enterobacter cloacae*	473	47	18	22	91	10	16	7
cAmpC (variants)								
ACT-type	32	3	1	1	30	0	0	1
pAmpC								
MIR-type	4	0	0	0	2	0	0	0
ESBL								
CTX-M/SHV-ESBL	5	3	0	0	3	0	0	0
ESBL + AmpC								
CTX-M + ACT/MIR-type	3	3	0	0	3	0	0	0
Carbapenemases								
OXA-48	3	0	0	1	2	0	0	0
AmpC + carbapenemases								
_P_AmpC/ACT-type + OXA48	5	0	1	2	3	0	1	0
_P_AmpC/ACT-type + VIM-1	1	1	1	1	1	1	1	1
ESBL + carbapenemases								
CTX-M + OXA-48	5	5	3	2	5	0	2	0
CTX-M + GES-6	1	1	0	0	1	0	0	0
CTX-M/SHV-BLEE + VIM-1	5	5	5	5	5	2	5	5
AmpC + ESBL + carbapenemases								
_P_AmpC/ACT-type + CTX-M + OXA-48	7	7	1	4	7	ND	1	ND
_P_AmpC/ACT-type + CTX-M + VIM-1	6	6	5	5	6	6	5	0
_P_AmpC/ACT-type + CTX-M + VIM-1 + OXA-48	1	1	1	1	1	1	1	0

cAmpC, chromosomal AmpC; C/T, ceftolozane/tazobactam; CZA, ceftazidime/avibactam; FEP, cefepime; IPM, imipenem; IPM/REL, imipenem/relebactam; MEM, meropenem; MEM/VAB, meropenem/vaborbactam; ND, not determined; pAmpC, plasmidic AmpC.

Regarding new β-lactam/β-lactamase inhibitor combinations, a total of 10, 16 and 7 isolates were resistant to ceftazidime/avibactam, imipenem/relebactam and meropenem/vaborbactam, respectively. The molecular analysis revealed that only one of these isolates carried a *bla*_ACT-type_ instead of a carbapenemase gene. The main carbapenemase found in these isolates was VIM-1 (*n* = 11) followed by OXA-48-like (*n* = 5) (Table 2). On the other hand, 91 isolates (19.2%) were resistant to ceftolozane/tazobactam, of which 32 isolates (35%) harboured an ACT-type or a plasmidic AmpC β-lactamase (MIR-type), 31 isolates (34%) harboured a carbapenemase and 6 isolates harboured an ESBL gene (Table 2). Regarding the remaining 22 isolates, no β-lactamase genes were found except the chromosomal *bla*_ACT_.

#### K. aerogenes

In the case of *K. aerogenes* only 5 of the 286 isolates (1.7%) showed resistance to cefepime. Of these, one isolate carried a plasmidic *ampC* gene, one isolate an NDM carbapenemase, and three isolates did not carry any acquired β-lactamase (Table 3). Ten isolates were resistant to imipenem and seven resistant to meropenem.

**Table 3.** Molecular profiles associated with resistant isolates of *Klebsiella aerogenes*

Microorganism	Total isolates (*n*)	FEP	IPM	MEM	C/T	CZA	IPM/REL	MEM/VAB
*Klebsiella aerogenes*	286	5	10	7	48	1	2	0
cAmpC (variants)								
ACT-type	1	0	0	0	1	0	0	0
pAmpC								
MIR-type	7	1	1	0	7	0	0	0
AmpC + carbapenemases								
_P_AmpC + NDM	1	1	1	1	1	ND	1	ND
ESBL + carbapenemases								
CTX-M + OXA-48	1	0	0	0	1	0	0	0

cAmpC, chromosomal AmpC; C/T, ceftolozane/tazobactam; CZA, ceftazidime/avibactam; FEP, cefepime; IPM, imipenem; IPM/REL, imipenem/relebactam; MEM, meropenem; MEM/VAB, meropenem/vaborbactam; ND, not determined; pAmpC, plasmidic AmpC.

Regarding new β-lactam/β-lactamase inhibitor combinations, all the isolates were susceptible to meropenem/vaborbactam, two isolates were resistant to imipenem/relebactam and only one was resistant to ceftazidime/avibactam (Table 3). Of these three isolates, only one carried a carbapenemase (NDM). On the other hand, 48 isolates (16.8%) were resistant to ceftolozane/tazobactam, of which only 7 isolates (14.5%) harboured a plasmidic AmpC β-lactamase (MIR-type) and 2 (4.1%) harboured a carbapenemase (Table 3). In the remaining 39 isolates only the chromosomal AmpC β-lactamase gene was found.

## Discussion

In this study, we reported data from the SMART surveillance study in Spain that focused on the activity of cefepime, carbapenems and new β-lactam/β-lactamase inhibitor combinations against *Enterobacter* spp. and *K. aerogenes.* Around 25% of the *E. cloacae* complex and around 35% of the *K. aerogenes* were resistant to third-generation cephalosporins. These data for *E. cloacae* complex are similar to those reported previously (24.3%), although they are slightly higher than those reported for *K. aerogenes* (23.3%).^17^ These resistance percentages have undergone variations over the years for both microorganisms, but without any clear trend (*P* > 0.05) (Table 1).

Regarding cefepime, differences have been observed among both kinds of microorganisms, with *E. cloacae* complex being more resistant than *K. aerogenes* (10% versus 2%). When the molecular characterization was performed, 15 of 47 (32%) cefepime-resistant *E. cloacae* isolates did not carry any β-lactamase, except for the chromosomal AmpC, suggesting that cefepime resistance also could be mediated by this β-lactamase or by another non-enzymatic mechanism, such as porin loss, as has been previously reported.^9,18,19^

Resistance to carbapenems was variable (1%–14%) in *E. cloacae* complex and *K. aerogenes* isolates, with meropenem being the most active carbapenem in both microorganisms (Table 1). Molecular characterization found the presence of at least one carbapenemase in most of them. A recent study has reported a worrying increase in carbapenemase-producing *E. cloacae* in the south of Spain from 2014 to 2022.^20^ However, based on our data we did not observe an increase in the carbapenem resistance percentage. This apparent discrepancy may be due to this increment occurring only in Andalusia, and not in the rest of the country, from where a large proportion of our data was collected.

Finally, resistance to new β-lactam/β-lactamase inhibitor combinations in *E. cloacae* complex and *K. aerogenes* was low in general, except for ceftolozane/tazobactam (Table 1). It is known that ceftolozane/tazobactam has lower activity in *E. cloacae* complex and *K. aerogenes* when AmpC β-lactamase is hyperproduced.^13^ This is because tazobactam inhibits AmpC β-lactamases less efficiently.^8^ In the case of ceftazidime/avibactam, only 11 of 759 isolates (1.4%) (10 *E. cloacae* complex and 1 *K. aerogenes*) showed resistance, of which all except 2 (1 *E. cloacae* and 1 *K. aerogenes*) carried a VIM-1 carbapenemase (Tables 2 and 3). The remaining two isolates did not carry any acquired β-lactamase, except the chromosomal AmpC. Resistance to ceftazidime/avibactam mediated by AmpC β-lactamases has been reported previously, but only in one study with one isolate from a patient treated with cefepime.^21^ Further studies are therefore needed to determine whether this β-lactamase is involved in resistance to this antibiotic. For imipenem/relebactam and meropenem/vaborbactam the situation was similar to ceftazidime/avibactam. From 18 (2.3%) isolates resistant to imipenem/relebactam and from 7 isolates resistant to meropenem/vaborbactam, only 1 *K. aerogenes* and 1 *E. cloacae* complex isolate, respectively, did not have any carbapenemase gene. Thus, ceftazidime/avibactam, imipenem/relebactam and meropenem/vaborbactam showed high activity in those isolates that do not carry any carbapenemase. Resistance to ceftazidime/avibactam not due to carbapenemases has already been reported in *E. cloacae*.^22,23^ This resistance was mainly associated with one or more amino acid deletions in helix H10 of the AmpC β-lactamase. However, to our knowledge, there are no studies describing the associated resistance mechanisms to imipenem/relebactam and meropenem/vaborbactam. Further studies are therefore needed to clarify this issue.

This study has one main limitation, i.e the molecular characterization was based on resistance to only three antibiotics (imipenem, imipenem/relebactam or ceftolozane/tazobactam), which may underestimate the real number of carbapenemase-producer strains.

In conclusion, there are differences in the microbiological characteristics between *E. cloacae* complex and *K. aerogenes*. In general, *E. cloacae* complex shows a more resistant profile than *K. aerogenes*, especially with cefepime. However, ceftazidime/avibactam, imipenem/relebactam and meropenem/vaborbactam combinations have high activity against both microorganisms. More studies are necessary to analyse the molecular mechanisms that drive resistance to these antibiotics.

## Supplementary Material

dlae087_Supplementary_Data

## Supplementary data

Table S1 is available as Supplementary data at *JAC-AMR* Online.

## References

[1] Antimicrobial Resistance Collaborators . Global burden of bacterial antimicrobial resistance in 2019: a systematic analysis. Lancet 2022; 399: 629–55. 10.1016/S0140-6736(21)02724-0 35065702 PMC8841637

[2] Cassini A, Högberg LD, Plachouras D et al Attributable deaths and disability-adjusted life-years caused by infections with antibiotic-resistant bacteria in the EU and the European economic area in 2015: a population-level modelling analysis. Lancet Infect Dis 2019; 19: 56–66. 10.1016/S1473-3099(18)30605-4 30409683 PMC6300481

[3] Tacconelli E, Cataldo MA, Dancer SJ et al ESCMID guidelines for the management of the infection control measures to reduce transmission of multidrug-resistant Gram-negative bacteria in hospitalized patients. Clin Microbiol Infect 2014; 20 Suppl 1: 1–55. 10.1111/1469-0691.12427 24329732

[4] Cantón R, Gottlieb T, Coombs GW et al Antimicrobial surveillance: a 20-year history of the SMART approach to addressing global antimicrobial resistance into the future. Int J Antimicrob Agents 2023; 62: 107014. 10.1016/j.ijantimicag.2023.107014 37866472

[5] Paterson DL, Rossi F, Baquero F et al In vitro susceptibilities of aerobic and facultative Gram-negative bacilli isolated from patients with intra-abdominal infections worldwide: the 2003 study for monitoring antimicrobial resistance trends (SMART). J Antimicrob Chemother 2005; 55: 965–73. 10.1093/jac/dki117 15849262

[6] Álvarez-Marín R, Navarro-Amuedo D, Gasch-Blasi O et al A prospective, multicenter case control study of risk factors for acquisition and mortality in *Enterobacter* species bacteremia. J Infect 2020; 80: 174–81. 10.1016/j.jinf.2019.09.017 31585192

[7] Tamma PD, Doi Y, Bonomo RA et al A primer on AmpC β-lactamases: necessary knowledge for an increasingly multidrug-resistant world. Clin Infect Dis 2019; 69: 1446–55. 10.1093/cid/ciz173 30838380 PMC6763639

[8] Tamma PD, Aitken SL, Bonomo RA et al Infectious Diseases Society of America guidance on the treatment of AmpC β-lactamase-producing Enterobacterales, carbapenem-resistant *Acinetobacter baumannii*, and *Stenotrophomonas maltophilia* infections. Clin Infect Dis 2022; 74: 2089–114. 10.1093/cid/ciab1013 34864936

[9] Barnaud G, Benzerara Y, Gravisse J et al Selection during cefepime treatment of a new cephalosporinase variant with extended-spectrum resistance to cefepime in an *Enterobacter aerogenes* clinical isolate. Antimicrob Agents Chemother 2004; 48: 1040–2. 10.1128/AAC.48.3.1040-1042.2004 14982805 PMC353102

[10] Fernández-Cuenca F, Rodríguez-Martínez JM, Martínez-Martínez L et al In vivo selection of *Enterobacter aerogenes* with reduced susceptibility to cefepime and carbapenems associated with decreased expression of a 40 kDa outer membrane protein and hyperproduction of AmpC β-lactamase. Int J Antimicrob Agents 2006; 27: 549–52. 10.1016/j.ijantimicag.2006.01.005 16697150

[11] Flury BB, Ellington MJ, Hopkins KL et al Association of novel nonsynonymous single nucleotide polymorphisms in ampD with cephalosporin resistance and phylogenetic variations in ampC, ampR, ompF, and ompC in *Enterobacter cloacae* isolates that are highly resistant to carbapenems. Antimicrob Agents Chemother 2016; 60: 2383–90. 10.1128/AAC.02835-15 26856839 PMC4808197

[12] Boattini M, Bianco G, Llorente LL et al Enterobacterales carrying chromosomal AmpC β-lactamases in Europe (EuESCPM): epidemiology and antimicrobial resistance burden from a cohort of 27 hospitals, 2020–2022. Int J Antimicrob Agents 2024; 63: 107115. 10.1016/j.ijantimicag.2024.107115 38367844

[13] Robin F, Auzou M, Bonnet R et al In vitro activity of ceftolozane-tazobactam against *Enterobacter cloacae* complex clinical isolates with different β-lactam resistance phenotypes. Antimicrob Agents Chemother 2018; 62: e00675-18. 10.1128/AAC.00675-18 29914962 PMC6125531

[14] Kim HJ, Fay MP, Feuer EJ et al Permutation tests for joinpoint regression with applications to cancer rates. Stat Med 2000; 19: 335–51 (correction: 2001; **20**: 655). 10.1002/(sici)1097-0258(20000215)19:3<335::aid-sim336>3.0.co;2-z 10649300

[15] Cantón R, Loza E, Arcay RM et al Antimicrobial activity of ceftolozane-tazobactam against Enterobacterales and *Pseudomonas aeruginosa* recovered during the study for monitoring antimicrobial resistance trends (SMART) program in Spain (2016–2018). Rev Esp Quimioter 2021; 34: 228–37. 10.37201/req/019.2021 33645948 PMC8179940

[16] García-Fernández S, Calvo J, Cercenado E et al Activity of imipenem/relebactam against Enterobacterales and *Pseudomonas aeruginosa* in Spain. SMART 2016–2020. Rev Esp Quimioter 2023; 36: 302–9. 10.37201/req/007.2023 36951688 PMC10238800

[17] Ye Y, Li JB, Ye DQ et al *Enterobacter* bacteremia: clinical features, risk factors for multiresistance and mortality in a Chinese university hospital. Infection 2006; 34: 252–7. 10.1007/s15010-006-5038-3 17033748

[18] Rodríguez-Martínez JM, Fernández-Echauri P, Fernández-Cuenca F et al Genetic characterization of an extended-spectrum AmpC cephalosporinase with hydrolysing activity against fourth-generation cephalosporins in a clinical isolate of *Enterobacter aerogenes* selected in vivo. J Antimicrob Chemother 2012; 67: 64–8. 10.1093/jac/dkr423 22001269

[19] Barnaud G, Labia R, Raskine L et al Extension of resistance to cefepime and cefpirome associated to a six amino acid deletion in the H-10 helix of the cephalosporinase of an *Enterobacter cloacae* clinical isolate. FEMS Microbiol Lett 2001; 195: 185–90. 10.1111/j.1574-6968.2001.tb10519.x 11179650

[20] Rivera-Izquierdo M . Alarming increase in hospital outbreaks of carbapenemase-producing *Enterobacter cloacae* in southern Spain. J Hosp Infect 2024; 145: 224–5. 10.1016/j.jhin.2023.11.008 38040035

[21] Shields RK, Iovleva A, Kline EG et al Clinical evolution of AmpC-mediated ceftazidime-avibactam and cefiderocol resistance in *Enterobacter cloacae* complex following exposure to cefepime. Clin Infect Dis 2020; 71: 2713–6. 10.1093/cid/ciaa355 32236408 PMC7744991

[22] Livermore DM, Mushtaq S, Doumith M et al Selection of mutants with resistance or diminished susceptibility to ceftazidime/avibactam from ESBL- and AmpC-producing Enterobacteriaceae. J Antimicrob Chemother 2018; 73: 3336–45. 10.1093/jac/dky363 30247546

[23] Lahiri SD, Giacobbe RA, Johnstone MR et al Activity of avibactam against *Enterobacter cloacae* producing an extended-spectrum class C β-lactamase enzyme. J Antimicrob Chemother 2014; 69: 2942–6. 10.1093/jac/dku237 24986496

