# Supplementary material for: Activity of cefepime, carbapenems and new β-lactam/β-lactamase inhibitor combinations on Enterobacter cloacae complex and Klebsiella aerogenes in Spain (SMART 2016–2022)
Source: JAC Antimicrob Resist. 2024 Jun 6;6(3):dlae087. doi: 10.1093/jacamr/dlae087 (PMC11154015; doi:10.1093/jacamr/dlae087)
Supplement: dlae087_Supplementary_Data [file dlae087_supplementary_data.docx]

**Supplementary material**

Table S1. Samples distribution from *E. cloacae* complex and *K. aerogenes* were isolated.

|  |  |
| --- | --- |
| **Samples** | **Total** |
| Blood | 76 |
| *Intra-abdominal* | 182 |
| - Abscess | 66 |
| - Appendix | 1 |
| - Colon | 2 |
| - Gall Bladder | 44 |
| - Liver | 3 |
| - Peritoneal Fluid | 66 |
| *Lower Respiratory Tract* | 366 |
| - Bronchial brushing | 41 |
| - Bronchoalveolar lavage | 44 |
| - Endotracheal aspirate | 170 |
| - Sputum | 111 |
| *Urinary Tract* |  |
| - Urine | 63 |
| *Other sources* | 53 |
| Total | 743 |
